# Neovessel as first manifestation of relapse of associated multifocal choroiditis and MEWDS

**DOI:** 10.1186/s40942-019-0175-x

**Published:** 2019-09-10

**Authors:** Eduardo Morizot, Camila Schiavo Froner

**Affiliations:** 1 Policlínica de Botafogo, Av. Pasteur, 72 - Botafogo, Rio de Janeiro, Brazil; 2 Institute Benjamin Constant, Av. Pasteur, 350 - Urca, Rio de Janeiro, Brazil

**Keywords:** MEWDS, Multifocal choroiditis, Neovascularization

## Abstract

**Purpose:**

To report a case of multifocal choroiditis (MC) that has relapsed as choroidal neovascularization in the contralateral eye followed by a mixed aspect of multiple evanescent white dot syndrome (MEWDS) and MC.

**Methods:**

Retrospective case report and literature review. The clinical findings were documented by fluorescein angiography, optical coherence tomography, and optical coherence tomography angiography (OCT-A).

**Results:**

The authors describe the case of a 39-year-old woman with prior ocular history of presumed MEWDS in her left eye, which developed into MC 7 years later in the same eye and 11 years later in the right eye, starting as choroidal neovascularization and developing into MEWDS. OCT-A showed neovessel in a supposedly active MC area outside the macular region in right and left eyes. OCT showed increased choroidal thickness in both eyes and a choroidal neovascularization in the right eye, treated using anti- VEGF therapy.

**Conclusion:**

This case corroborates the proximity of some inflammatory diseases such as MC and MEWDS. OCT-A has opened new horizons for the better understanding of some retinal diseases by providing more thorough and promising morphological analyses using enhanced tools.

## Introduction

Multifocal choroiditis (MC) is part of a spectrum of inflammatory diseases known as White Dot Syndrome. MC is characterized by yellowish-white lesions that might affect the posterior pole and mid-periphery and is more common in women [1–3]. In 23–25% of affected eyes, it develops into choroidal neovascularization [4].

Within this spectrum, we also find the multiple evanescent white dot syndrome (MEWDS), which affects mostly young myopic women, associated with photopsias and central or paracentral scotoma. Fluorescein angiography shows hyperfluorescent spots that generally converge circularly, with a discrete staining in the late phases of the exam. Optical Coherence Tomography (OCT) reveals loss of ellipsoid zone. The granular shaped stain practically confirms the diagnosis. These changes decrease within weeks or months, with a good prognosis for the patient [5, 6]. Multifocal choroiditis and MEWDS might sometimes coexist, and one precedes the other [7].

This study aims to report a case of multifocal choroiditis that has relapsed as choroidal neovascularization in the contralateral eye followed by a mixed aspect of MEWDS and MC. A consent form was read and explained to the patient and signed by her. This study was conducted in accordance with the Declaration of Helsinki.

## Case report

A 39-year-old woman presented with a two days complaint of paracentral scotoma in the right eye (R.E.) on August 15, 2017. Her ophthalmic history had been significant for MEWDS in this eye, diagnosed in 2006; however, she had no exams to confirm that. In 2013, she was diagnosed with subfoveal neovascular membrane with multifocal choroiditis scars around in the left eye (L.E.), and has been treated successfully since then with anti- VEGF, with an average of three injections per year.

**Fig. 1:**
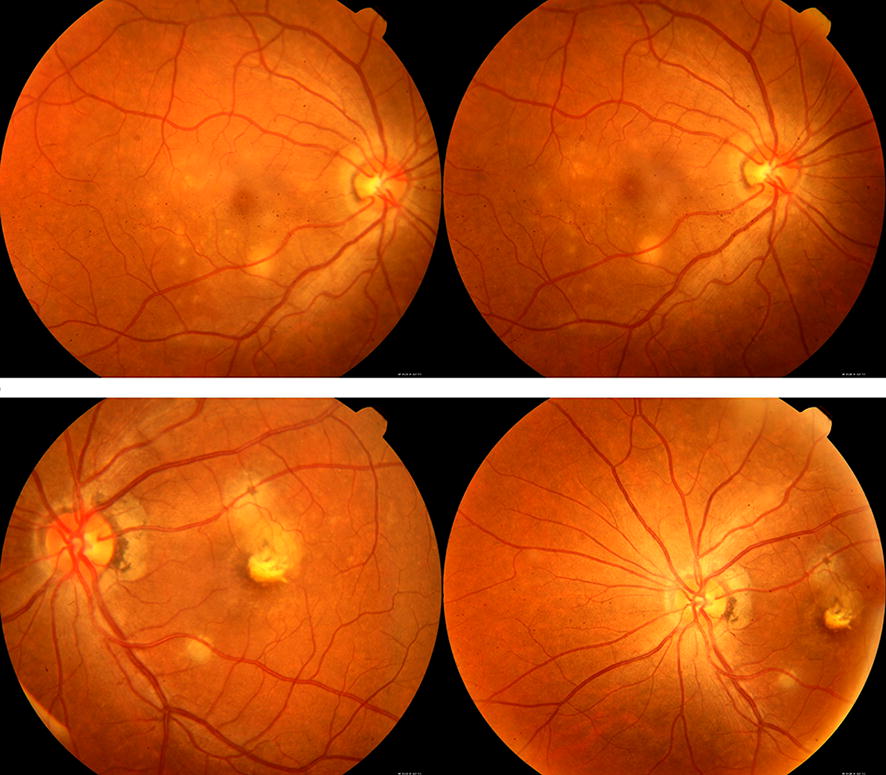
Sub-retinal neovascular membrane surrounded by atrophic pigmentary lesions in L.E. White-yellow lesion under the inferior vascular arcade in both eyes

Her visual acuity was 20/20 in the R.E. and 20/25 in the L.E. (under − 0.25 diopters in both eyes). Biomicroscopy, intraocular pressure, and pupil reflexes were normal, and there was mild pigment disturbance in the posterior pole of the R.E. fundus and a symmetrically deep round lesion under the inferior vascular arcade in both eyes. There were areas of fibrosis in the macula, due to the long period with neovascular membrane, surrounded by atrophic pigmentary scars in the left eye (Fig. 1). Fluorescein Angiography revealed a roundish hyperfluorescent lesion in both eyes corresponding to those observed in the fundus, with leakage in the R.E. and staining in the L.E. There was a foveal neovascular membrane in the latter, surrounded by hyperfluorescent scars.

**Fig. 2:**
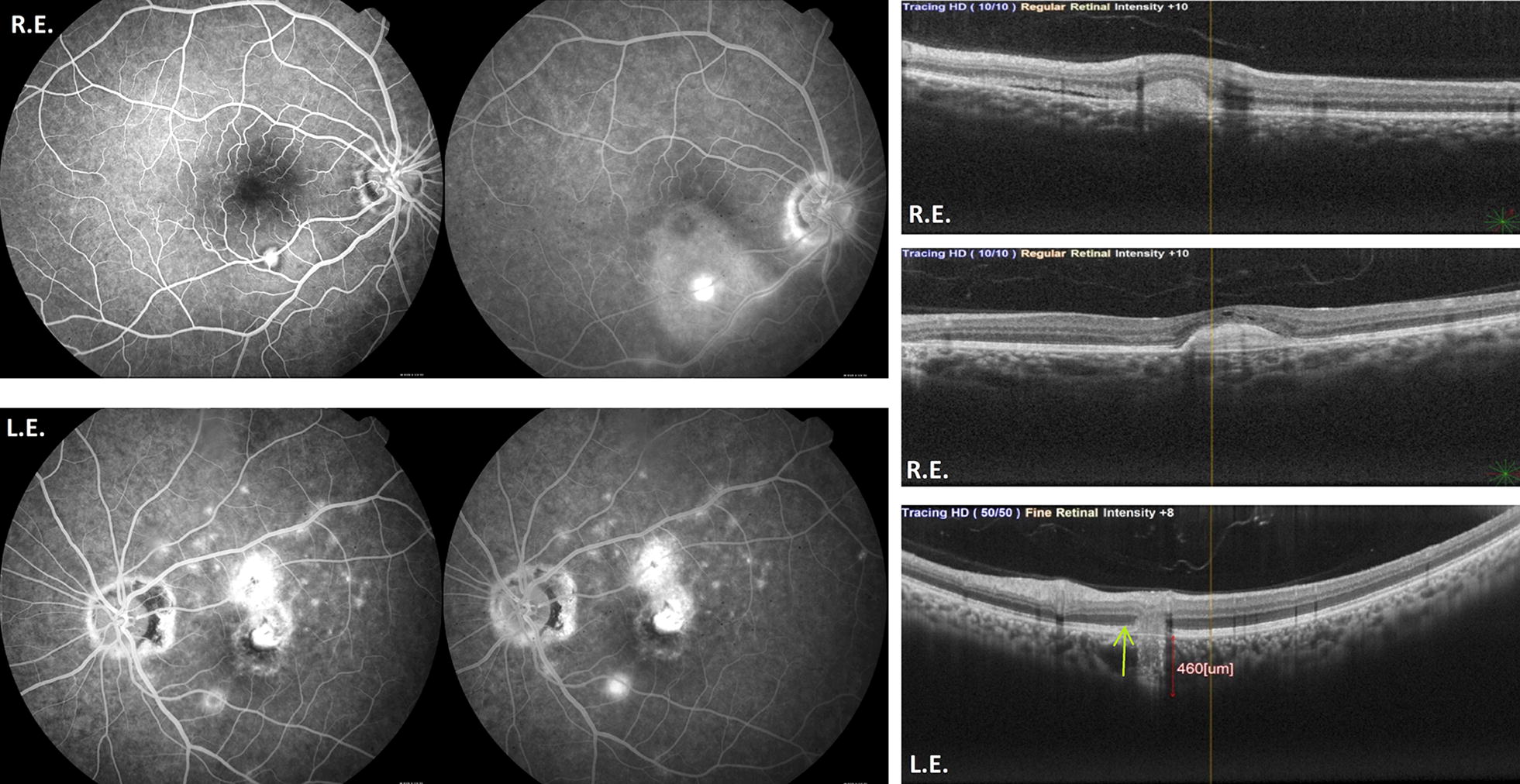
OCT-In the R.E.: hyperreflective lesion above the RPE in the inferior arcade, followed superiorly by subretinal liquid, with increased choroidal thickness in the posterior pole. In the L.E.: hyperreflective lesion above the RPE of the macular area and hyperreflectivity throughout the outer segment and choroid of the inferior arcade, with increased choroidal thickness in this area

**Fig. 3:**
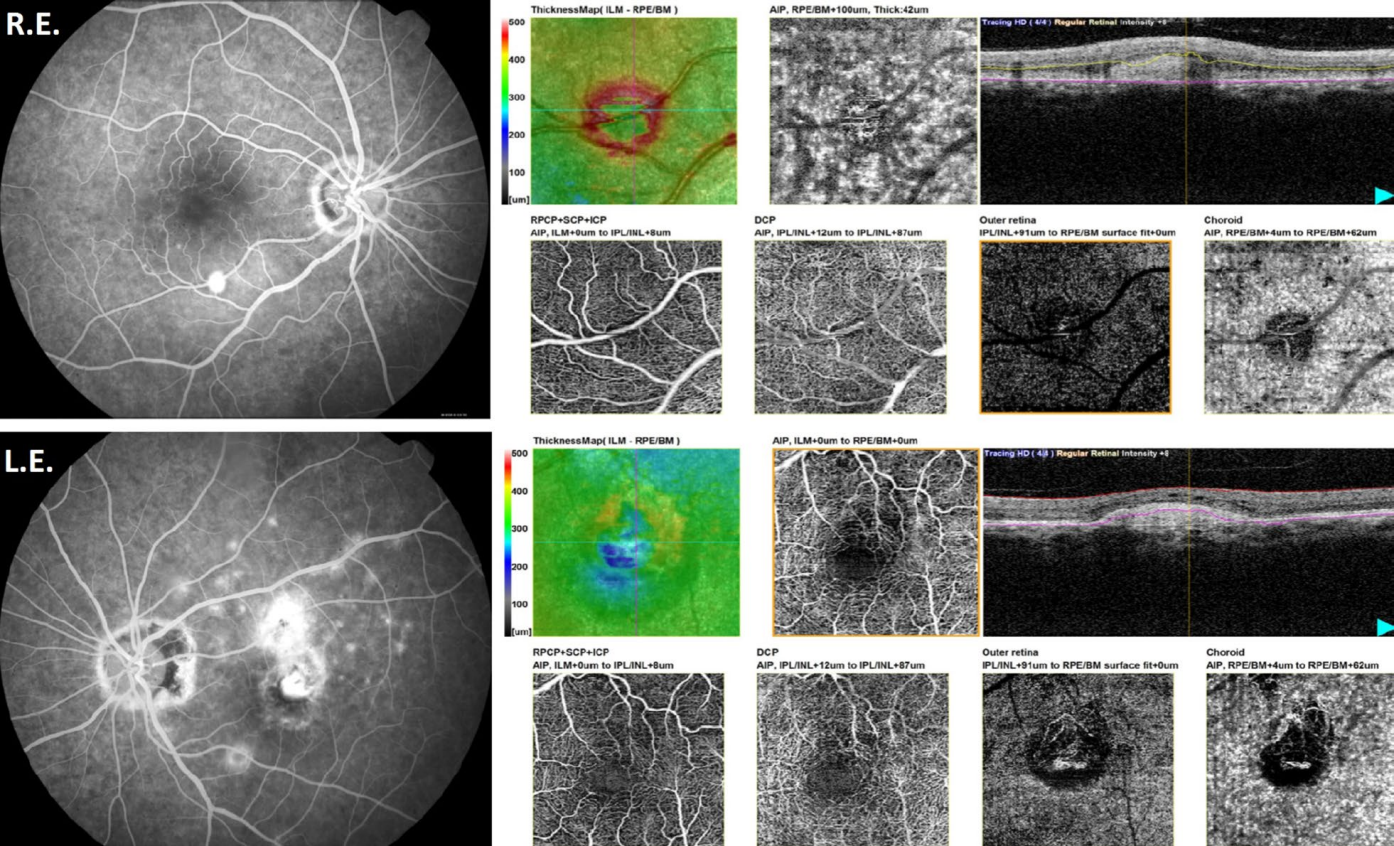
OCT-A showing neovessel corresponding to the round lesion in the inferior arcade in R.E. and a foveal membrane in the L.E

OCT (Spectral domain optical coherence tomography—Spectralis; Heidelberg Engineering, Heidelberg, Germany) showed a hyperreflective lesion above the retinal pigment epithelium (RPE) in the inferior arcade of the R.E., followed superiorly by subretinal liquid, with increased choroidal thickness in the posterior pole. In the L.E., there was also a hyperreflective lesion above the RPE of the macular area and a hyperreflectivity throughout the outer segment and choroid of the inferior arcade, with an increased choroidal thickness in this area (Fig. 2). The OCT-angiography (OCT-A) revealed a neovessel corresponding to the round lesion in the inferior arcade in both eyes and a foveal membrane in the L.E. (Fig. 3). Serological examinations for inflammatory diseases were requested and she was treated with intravitreal anti-VEGF.

On August 23, 2017 she returned complaining about blurred vision in the R.E. She had normal results for inflammatory diseases but a high level of Toxoplasmosis IgG (1/400). The fundus was unchanged except for a mild vitritis. She was treated with sulfamethoxazole and prednisone, but the treatment was suspended 3 days later when she had a positive pregnancy test.

**Fig. 4:**
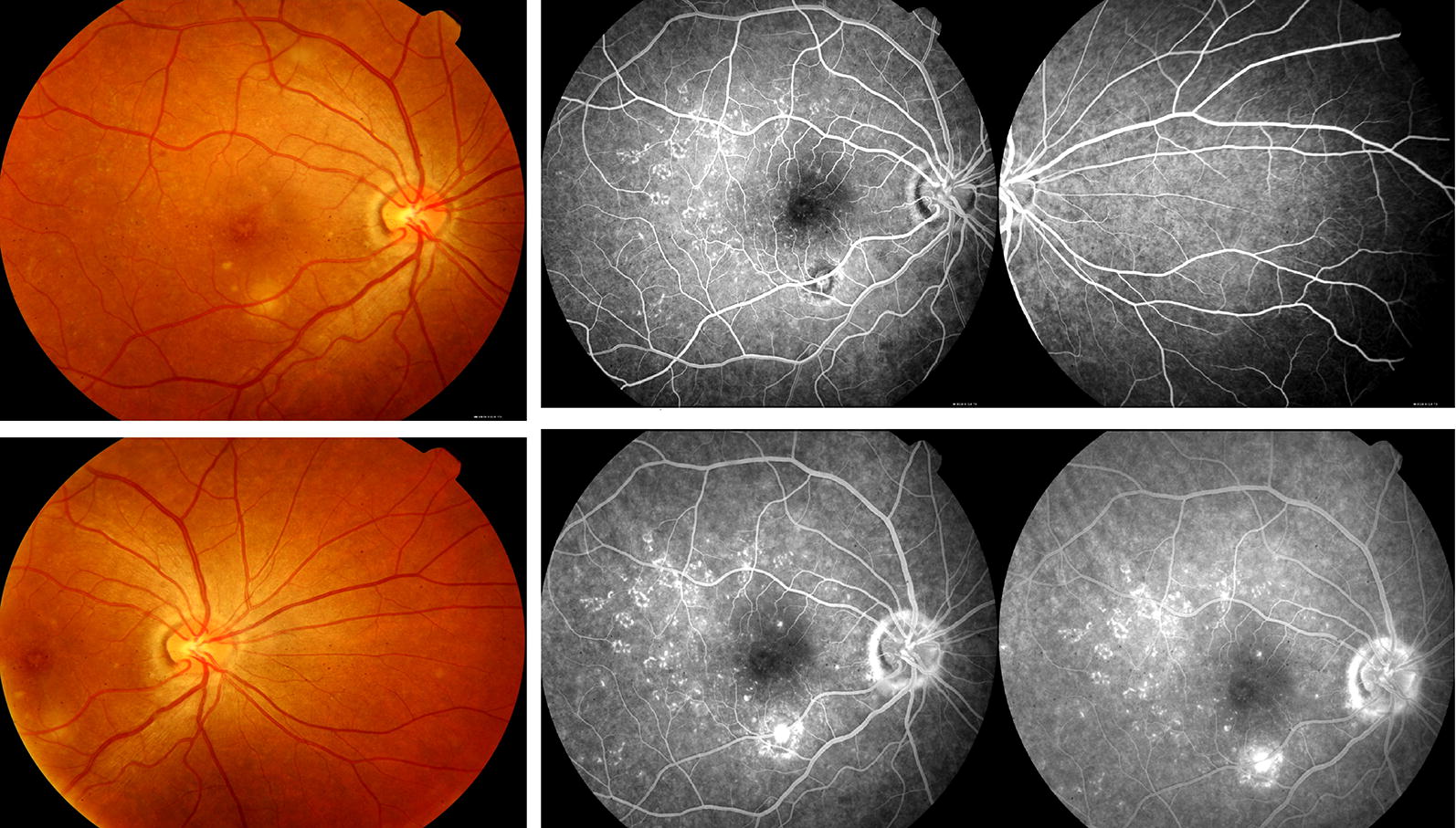
Hyperfluorescent spots arranged in circles with mild staining in the late phase

On August 29, 2017, she complained of mildly blurred vision in the R.E. Visual acuity was 20/25 in the R.E. and there were white dots in the posterior pole of the fundus. The inferior temporal lesion had a dry aspect. The fluorescein angiography revealed hyperfluorescent spots in the posterior pole, arranged in circles in the temporal region, with mild staining in the late phase. The inferior temporal lesion was also stained, without leakage (Fig. 4). One month later, her visual acuity was 20/20 in the R.E., and there was ellipsoid zone interruption beside the temporal inferior membrane, without subretinal liquid in her OCT examination.

**Fig. 5:**
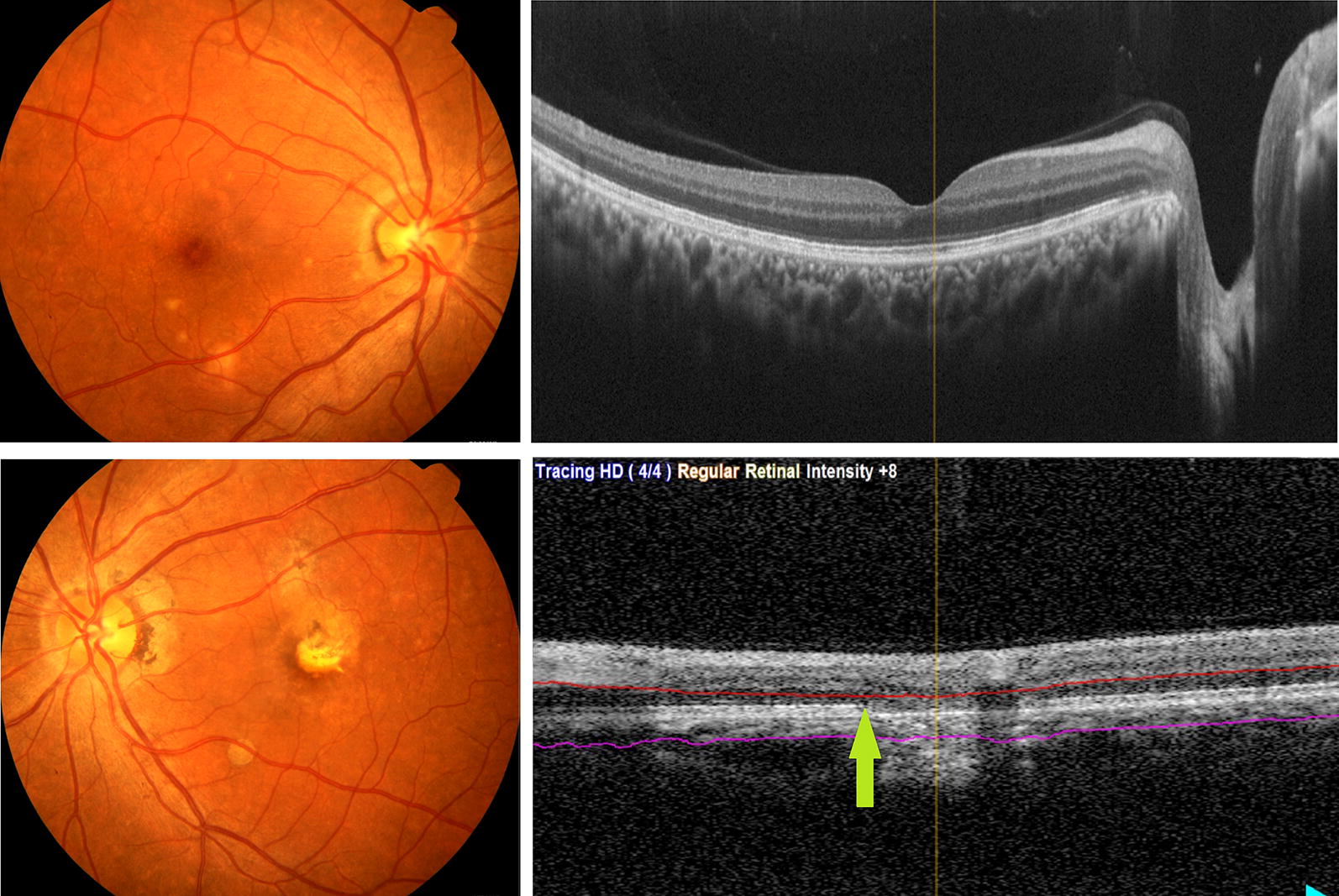
White dot spots, granulate macula and ellipsoid zone reconstitution

She returned on October 23, 2017 without any symptoms. Visual acuity in R.E. was 20/20, and there were white dots in the posterior pole of the fundus and macular granularity. OCT showed ellipsoid zone reconstitution in the R.E. But there was still with a hyperreflectivity area in the outer retina and choroid in the inferior temporal arcade of the L.E. (Fig. 5).

On December 15, 2017, four months after baseline, her visual acuity was 20/20 in the R.E. and 20/25 in the L.E. with no new symptoms.

## Discussion

Idiopathic multifocal choroiditis is a generic term which encompasses three diseases. Punctate inner choroiditis (PIC), characterized for being limited to the posterior pole with no presence of cellularity in the vitreous and anterior chamber; Multifocal Choroiditis associated with Panuveitis, combined with the presence of lesions affecting the posterior pole and/or mild periphery; and Progressive Subretinal Fibrosis, with fibrosis formation, disease progression and a worse prognosis than the others [8]. The two first forms are minor forms of multifocal choroiditis, and the latter one is the major form.

In literature, approximately 30% of patients with multifocal choroiditis with panuveitis developed into choroidal neovascularization [9]. Zahid et al. [10] analyzed 18 eyes with chorioretinal lesions due to multifocal choroiditis using OCT-A, and found the presence of neovascularization inside the subretinal lesions in 91% of them and mixed lesions (subretinal and subpigment epithelium) in 100%. There were no neovessels in two patients with active multifocal choroiditis below the pigment epithelium.

The presence of neovascular membrane in MEWDS is not common. Fernandéz-Barriento et al. [11] reported choroidal neovascularization 13 years after an episode of MEWDS. Papadia et al. [12] described a case where subretinal neovascular membrane preceded the onset of MEWDS.

Choroidal neovascularization preceded the onset of chorioretinal inflammatory diseases in four of 58 patients studied with idiopathic subretinal neovascular membrane. Among them, two showed compatible condition with MEWDS in the contralateral eye; one case developed into multifocal choroiditis and the other developed into inner punctate choroiditis in the ipsilateral eye [13].

In four atypical cases of MEWDS, with presence of yellowish foveal exudates, there was increased choroidal thickness and choroidal neovascularization diagnosed at presentation or during the follow-up, detected with fluorescein angiography, indocyanine green, and OCT-A [14].

In the present case, the submacular neovascular membrane in the L.E. appeared seven years after presumed MEWDS, but with typical multifocal choroiditis lesions, and 11 years later in the R.E. inside the chorioretinal lesion outside the macular area. This finding was confirmed by fluorescein angiography and OCT-A, and was quickly inactivated with anti-VEGF. OCT also showed increased choroidal thickness. Interestingly, the L.E. showed a reactivated scar symmetrical to the contralateral eye without exhibiting leakage.

Two weeks after the beginning of the symptoms, Fluorescein Angiography showed hyperfluorescent spots in the R.E., arranged in circles, focal loss of ellipsoid zone (which was re-established in the follow-up), and granulate macula, thus characterizing MEWDS. Four months later, already without any sign of activity, there were yellowish atrophic scars in the posterior pole compatible with multifocal choroiditis, closing the inflammatory cycle.

## Conclusion

Idiopathic multifocal choroiditis and MEWDS probably belong to the same disease spectrum, and are even closer to each other than other diseases within the White Dot Syndrome, maybe mediated by an immune process which would trigger one or another, or both.

OCT-A has opened new horizons, paving a new path to the morphological analysis of retinal diseases, with potential to change the paradigms of some conditions. Using this exam and performing multimodal examinations, other case reports might contribute with a better understanding of these diseases.
